# Environmental Adaptation for Person-Centered Care: Lessons from an Underground Medical Facility

**DOI:** 10.1093/geroni/igaf122.3258

**Published:** 2025-12-31

**Authors:** Sharon Ost-Mor, Elena Titelman, Eduard Zlyesov, Inna Shugaev

**Affiliations:** University of Haifa, Haifa, HaZafon, Israel; Fliman Medical Center, Haifa, HaZafon, Israel; Fliman Medical Center, Haifa, HaZafon, Israel; Fliman Medical Center, Haifa, HaZafon, Israel

## Abstract

Environmental factors significantly impact health outcomes as people age, yet little research explores strategies for maintaining quality care when traditional healthcare environments become unavailable. This study examines the successful conversion of an underground parking facility into a functional geriatric rehabilitation center during an extended emergency relocation at Fliman Medical Center. Through a detailed case study, including in depth interviews and diaries, the research analyzes key environmental modifications and operational adaptations necessary to sustain comprehensive carein a non-traditional setting. Specifically, it evaluates environmental control systems, space utilization, therapeutic environment creation, and strategies for preserving patient and staff wellbeing. Findings highlight the effective implementation of critical adaptations, including optimized lighting, ventilation, and temperature control tailored to rehabilitation needs; efficient spatial organization to support rehabilitation services; the creation of therapeutic environments despite spatial constraints; and innovative solutions to maintain patient and staff wellbeing under suboptimal conditions. These results demonstrate that high-quality medical care can be preserved in unconventional settings through strategic environmental modifications. This study offers valuable insights for healthcare facility design, emergency preparedness planning, and the adaptation of non-traditional spaces for care delivery, providing practical guidance for maintaining older adults’ quality care during emergencies and facility transitions.

